# Case–control study of behavioural and societal risk factors for sporadic SARS-CoV-2 infections, Germany, 2020–2021 (CoViRiS study)

**DOI:** 10.1017/S0950268824000050

**Published:** 2024-01-15

**Authors:** Bettina M. Rosner, Gerhard Falkenhorst, Isabella Kumpf, Maren Enßle, Andreas Hicketier, Achim Dörre, Klaus Stark, Hendrik Wilking

**Affiliations:** Department of Infectious Disease Epidemiology, Robert Koch Institute, Berlin, Germany

**Keywords:** public health, infectious disease epidemiology, COVID-19, SARS-CoV-2, virus infection

## Abstract

During the COVID-19 pandemic in Germany, a variety of societal activities were restricted to minimize direct personal interactions and, consequently, reduce SARS-CoV-2 transmission. The aim of the CoViRiS study was to investigate whether certain behaviours and societal factors were associated with the risk of sporadic symptomatic SARS-CoV-2 infections. Adult COVID-19 cases and frequency-matched population controls were interviewed by telephone regarding activities that involved contact with other people during the 10 days before illness onset (cases) or before the interview (controls). Associations between activities and symptomatic SARS-CoV-2 infection were analysed using logistic regression models adjusted for potential confounding variables. Data of 859 cases and 1 971 controls were available for analysis. The risk of symptomatic SARS-CoV-2 infection was lower for individuals who worked from home (adjusted odds ratio (aOR) 0.5; 95% confidence interval (CI) 0.3–0.6). Working in a health care setting was associated with a higher risk (aOR: 1.5; 95% CI: 1.1–2.1) as were private indoor contacts, personal contacts that involved shaking hands or hugging, and overnight travelling within Germany. Our results are in line with some of the public health recommendations aimed at reducing interpersonal contacts during the COVID-19 pandemic.

## Introduction

The World Health Organization (WHO) declared a COVID-19 pandemic caused by SARS-CoV-2 in March 2020 [1, 2]. In the early phases of the pandemic, vaccinations were not available and medical treatment options were limited. Therefore, countries implemented a variety of non-pharmaceutical interventions (NPIs) to limit person-to-person contacts and thereby reduce the number of new SARS-CoV-2 infections.

In Germany, public health measures strongly affected various societal activities, for example, the closure of schools, restaurants/bars, and other venues where people get together, or restricting the number of participants in social gatherings. In addition, wearing face masks became mandatory in many situations, for example, when shopping in stores, using public transport, or visiting patients in hospitals and nursing homes. As general precautions, the Ministry of Health and public health authorities at national, state, and local levels promoted keeping physical distance to other people (at least 1.5 m), wearing face masks when in contact with other people, adhering to general hygienic measures (washing hands frequently and following good sneezing and coughing etiquette), and ensuring adequate ventilation in rooms with people [3, 4]. In the beginning of the pandemic, when we planned this study, it was not clear which personal behaviours and activities might be associated with SARS-CoV-2 infections. The goal of the CoViRiS (*Corona-Virus Risiko-und Schutzfaktoren*) study was to determine the associations of a variety of behavioural and societal factors with the risk for sporadic symptomatic SARS-CoV-2 infections.

## Methods

### Study design

Our study was designed as a case–control study. As cases we recruited adults with symptomatic SARS-CoV-2 infections (diagnosed by PCR) who were notified to a local health authority in Germany between November 2020 and November 2021 (sporadic cases only). As controls we recruited adults from the general population that were frequency-matched to cases based on age group (in 10-year intervals), sex, and district of residence (same or neighbouring districts). By frequency-matching the distribution of these three matching variables was intentionally made similar in the case group and in the control group. Our aim was to recruit 1 200 case-patients and 2 400 control-persons (1:2 ratio). Case-patients and controls were interviewed by telephone mainly about certain activities conducted in the 10 days before disease onset (case-patients) or before the interview (controls), respectively.

### Recruitment of study participants

Local health authorities supported the recruitment of notified COVID-19 cases for the study on a voluntary basis. They provided the Robert Koch Institute (RKI) with contact information of COVID-19 cases who verbally agreed to participate. Contact information of case-patients who subsequently agreed also in writing was forwarded from the RKI to a social and market research institute (USUMA GmbH, Berlin, Germany) commissioned by the RKI to schedule and conduct telephone interviews with case-patients as well as controls. Potential controls were contacted by USUMA using random digit dialling. Eligibility criteria for both groups were: at least 18 years of age; not living in a nursing home; no travelling abroad within the 10 days before disease onset (case-patients), or before the interview (controls); no professional health care activities where direct contact with COVID-19 patients without adequate personal protection equipment occurred. In addition, case-persons were only eligible if they were sporadic cases (not part of a local outbreak, and infection through household transmission unlikely). To identify non-sporadic cases, we checked if COVID-19 cases were notified as part of a local outbreak in our surveillance system (Surv*N*et@RKI). Notified outbreak cases were not invited to participate in our study. In addition, cases who verbally expressed an interest in study participation were sent a short screening questionnaire together with general study information. Cases who reported contact with a SARS-CoV-2 positive person living in the same household before onset of their own symptoms were also defined as non-sporadic cases (infection likely obtained through household transmission) and excluded from study participation. Controls were only eligible if they had never tested positive for SARS-CoV-2 infection.

### Telephone interviewing

Computer-assisted telephone interviewing was conducted using a structured, standardized questionnaire. We asked about various factors and activities that involved personal contact with other people (e.g., contacts at work, private contacts, and visiting restaurants and bars). In addition, we queried certain pre-existing medical conditions and some sociodemographic characteristics. We asked study participants to rank themselves on a social status scale between 1 and 10, where ‘10’ meant people with the most money, the highest degree of education, the best jobs, and ‘1’ meant people with the least money, the lowest degree of education, the worst jobs or no jobs [5]. We queried if study participants had been vaccinated against COVID-19 after vaccinations became more widely available in January 2021. Case-patients and controls were interviewed between 8 December 2020 and 30 November 2021.

### Statistical analyses

We identified an adjustment set of five potential confounding factors of the hypothesized association of each exposure variable with the outcome symptomatic SARS-CoV-2 infection. We determined the adjusted odds ratio (aOR) for each exposure variable with 95% confidence interval (CI) using logistic regression models with this adjustment set. The five adjusting variables were: (i) age group (four categories); (ii) sex (female/male); (iii) urban or rural type of district of residence (four categories; variable ktyp4 [6]); (iv) vaccination status (yes/no); and (v) 7-day SARS-CoV-2 incidence (number of reported new infections in the past 7 days per 100 000 population) in the district of residence on day 2 before disease onset (case-patients) or on the day of the interview (controls) (five categories) (see Table 1 for details). Age group, sex, and urban/rural type of the district of residence were matching variables and included in the adjustment set to account for residual confounding. Vaccination status was identified as a confounder in our models because we assumed that vaccinated and unvaccinated individuals might have behaved differently. Vaccinated individuals might have been more socially active than unvaccinated individuals because they considered themselves protected from SARS-CoV-2 infection. Case-patients and controls who were interviewed before January 2021 were categorized as not vaccinated. The 7-day incidence in the district of residence was included in the adjustment set to account for confounding due to small-scale geographical and temporal variations of the SARS-CoV-2 infection risk, which might have differed for case-patients and controls. Country of birth and degree of school education were not originally included in our models because we had not observed associations of these factors with SARS-CoV-2 infections in adjusted single-variable analyses. We repeated our analyses of associations with modified logistic regression models that included country of birth and degree of school education in addition to the five adjusting variables mentioned above. We also performed data analyses with a subset of 325 case-patients and 1 083 controls with disease onset or interview dates, respectively, in the third wave (week 9/2021 to week 37/2021) of the pandemic in Germany [7], because the timing of case and control interviews was more closely aligned in the third wave, and, presumably, NPIs were more homogenous across regions in this limited time period. Statistical significance of estimated associations was assessed using Wald tests. *P*-values <0.05 were considered as an indication of statistical significance. We used Stata 17 (Stata Corporation LLC, College Station, TX, USA) for statistical analyses.

**Table 1.** Characteristics of the study population, case–control study, Germany, 2020–2021

	Case-patients (%, *N* or *n*/*N* with respective data)	Control-persons (%, *N* or *n*/*N* with respective data)
Total	100% (859)	100% (1 971)
Sex		
Female	57.8% (496/858)	54.7% (1 079/1 971)
Male	42.2% (362/858)	45.3% (892/1 971)
Age		
Median (range)	47 years (18–83 years)	49 years (18–93 years)
Age group (years)		
18–29	17.1% (147/859)	13.5% (266/1 965)
30–49	37.5% (322/859)	39.4% (774/1 965)
50–69	40.3% (346/859)	50.0% (805/1 965)
70+	5.1% (44/859)	6.1% (120/1 965)
Type of district of residence		
Large urban municipality	24.6% (211/858)	23.1% (455/1 971)
Urban district	40.2% (345/858)	40.9% (807/1 971)
Rural district with some agglomeration	22.4% (192/858)	22.7% (447/1 971)
Sparsely populated rural district	12.8% (110/858)	13.3% (262/1 971)
Vaccination status		
Not vaccinated	73.1% (621/849)	59.6% (1 171/1 965)
Vaccinated at least once	26.9% (228/849)	40.4% (794/1 965)
7-day incidence in district of residence^a^ (cases/100 000 population)		
≤25	5.7% (48/849)	20.4% (401/1 965)
26–50	11.2% (95/849)	12.1% (237/1 965)
51–100	43.6% (370/849)	20.3% (399/1 965)
101–250	38.2% (324/849)	38.8% (763/1 965)
>250	1.4% (12/849)	8.4% (165/1 965)
Degree of school education (years of school education)		
Secondary school certificate (‘Hauptschule’; 9 or 10 years) or no degree or no degree yet	11.7% (100/858)	11.8% (231/1 954)
Secondary school certificate (‘Realschule’; 10 years)	34.0% (292/858)	29.7% (581/1 954)
High school diploma/A-levels (‘Abitur’; 12 or 13 years)	54.3% (466/858)	58.4% (1 142/1 954)
Self-perceived social status on a scale of 1–10		
1–4	7.3% (62/846)	10.8% (206/1 909)
5–7	73.9% (625/846)	71.8% (1 370/1 909)
8–10	18.8% (159/846)	17.4% (333/1 909)
Country of birth		
Germany	90.7% (779/859)	91.2% (1 794/1 968)
Other country	9.3% (80/859)	8.8% (174/1 968)
Citizenship		
German or German plus other	96.3% (827/859)	96.6% (1 902/1 968)
Other	3.7% (32/859)	3.4% (66/1 968)
Native language		
German or German plus other	92.8% (797/859)	93.0% (1 830/1 968)
Other	7.2% (62/859)	7.0% (138/1 968)

a Seven-day incidence in the district of residence on day 2 before disease onset (case-patients) or on the day of interview (controls).

### Data protection

The study design was approved by the data protection officer of the Robert Koch Institute. Data protection measures included adherence to EU’s general data protection regulation. Collected data were recorded anonymously.

### Ethics approval

Ethics approval was granted from the ethics committee of the Charité University Medicine, Berlin, Germany (EA4/162/20; 14 August 2020). The study was conducted in accordance with relevant guidelines and regulations.

### Consent to participate

Participation in the study was voluntary. Informed consent was obtained from all subjects who participated in the study. Case-patients gave verbal and written consent before the telephone interview. Controls gave verbal consent before the telephone interview.

## Results

### Participation of case-patients and controls in the study

Of 3 297 case-patients who verbally expressed an interest in participating in our study, 859 completed an interview, resulting in an overall response rate of 26%. Due to constraints in the recruitment process, the number of eligible notified COVID-19 patients who were not approached by the local health authorities or who were invited but declined participation in our study remained undetermined. Fewer case-patients could be included in our study than originally planned. The main reason for non-participation, despite initial interest, was the failure to return the written consent form by paper post (*n* = 1 771; 54%) (Supplementary Figure S1). Case-patients were interviewed, on average (median), 32 days after disease onset (25th to 75th percentile (IQR): 24–43 days). Anonymized data of 1 971 interviewed control-persons were included in data analyses.

### Sociodemographic and other characteristics of case-patients and controls

Case-patients and controls were similar with respect to the matching criteria age group, sex, and residence in urban or rural districts. Median age was 47 years for case-patients and 49 years for controls. Both groups were also similar regarding their highest degree of school education (Table 1).

### Symptoms and disease onset of COVID-19 among case-patients

The most frequently reported symptoms were a runny nose (65%), coughing (65%), and general symptoms of illness (91%) such as tiredness/fatigue, and headache. More than half of case-patients reported loss of smell (54%) or taste (53%), and about 40% reported fever (body temperature of 38.5°C or higher). Shortage of breath or dyspnoea was reported by 37% of case-patients, and 3% had pneumonia. Three per cent were hospitalized because of COVID-19; the median duration in hospital was 6 days (range: 1–24 days; IQR: 2–15 days). Three of 859 case-patients had to be treated in an intensive care unit; two of those had to be ventilated. Disease onsets of most case-patients were in the second and third wave of the COVID-19 pandemic in Germany (40% and 38%, respectively). The second wave was dominated by wild-type SARS-CoV-2 (Wuhan strain), the third wave by the variant of concern (VOC) Alpha (B.1.1.7). A smaller percentage of case-patients became infected in the phase between the third and fourth wave (3%) or in the fourth wave (19%), which was dominated by VOC Delta (B.1.617.2) [7].

### Associations of exposures with symptomatic SARS-CoV-2 infections

Associations of single exposures were investigated with logistic regression models including adjustment for five covariables as described above. Because of the number of exposures that we queried, we present the results of our logistic regression analyses according to topic. Adding the variables country of birth and degree of school education (three categories) (Table 1) to our models as additional adjustment factors did not have an impact on our results (data not shown). When we limited our analyses to a subset of cases and controls in the third wave of the pandemic in Germany, the results did not differ substantially compared with the complete dataset (Supplementary Tables S7–S12). Small differences in the results may be due to the decrease in power to detect associations with the smaller dataset.

#### Household

**Figure 1.**
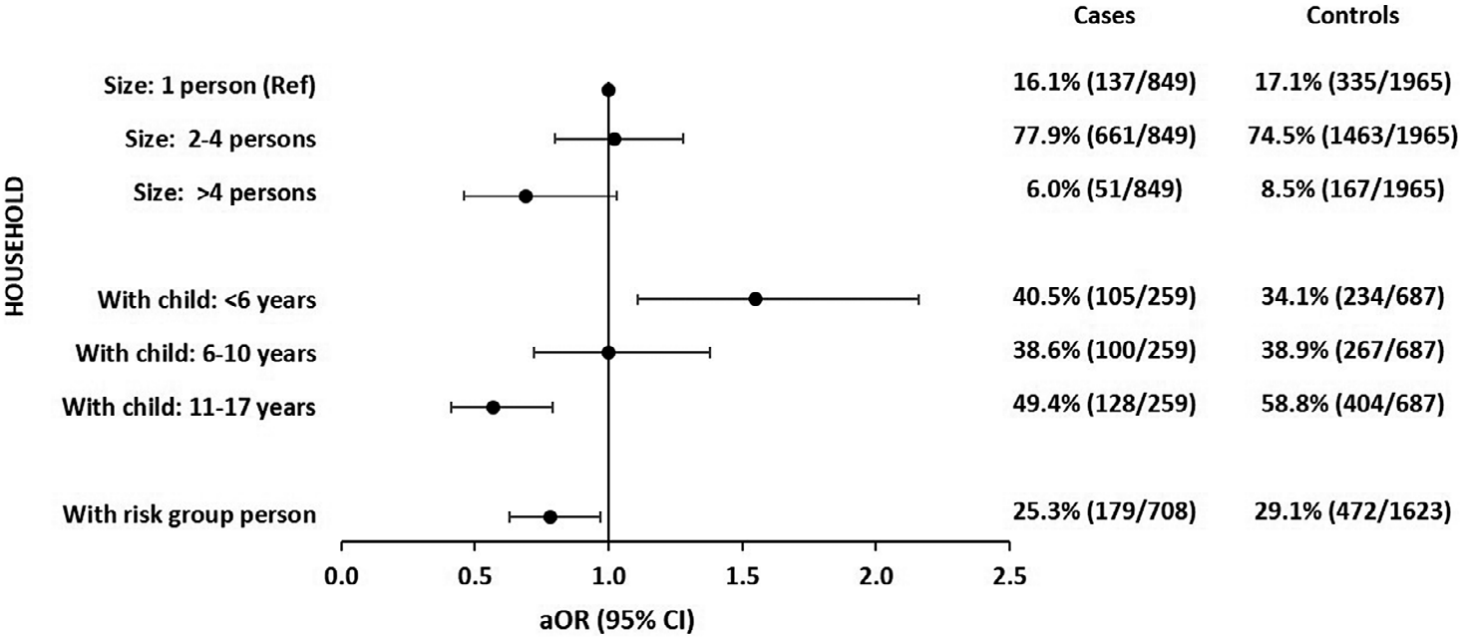
Adjusted odds ratios with 95% confidence intervals of associations of household characteristics with symptomatic SARS-CoV-2 infections. Each exposure (household size (three categories); household with child/children of a certain age group (two categories: yes/no); household with a risk group person (two categories: yes/no)) was analysed in a logistic regression model with five adjusting covariables (see the ‘Methods’ section for details). Analysis of the association with children’s age was restricted to participants living with at least one child <18 years of age (259 cases; 687 controls). Analysis of the association with living with a ‘risk group person’ was restricted to participants not living alone (708 cases; 1 623 controls). Examples of ‘risk group person’: person>60 years of age or/and with chronic disease. Observations with missing values in the variable of interest or any of the five adjusting covariables were excluded from analysis.

Compared with persons living alone, the risk of symptomatic SARS-CoV-2 infection was not increased for persons living together with others. However, among those living in households with at least one child, risk of symptomatic SARS-CoV-2 infection was increased if the child was <6 years of age (aOR: 1.6; 95% CI: 1.1–2.2), neither increased nor decreased if the child was 6–10 years of age (aOR: 1.0; 95% CI: 0.7–1.4), and decreased if the child was 11–17 years of age (aOR: 0.6; 95% CI: 0.4–0.8). We observed a negative association with symptomatic SARS-CoV-2 infections if a person belonging to a COVID-19 risk group, that is, a person with a chronic disease or a person 60 years or older, lived in the household (aOR: 0.8; 95% CI: 0.6–1.0) (Figure 1 and Supplementary Table S1).

#### Work environment

**Figure 2.**
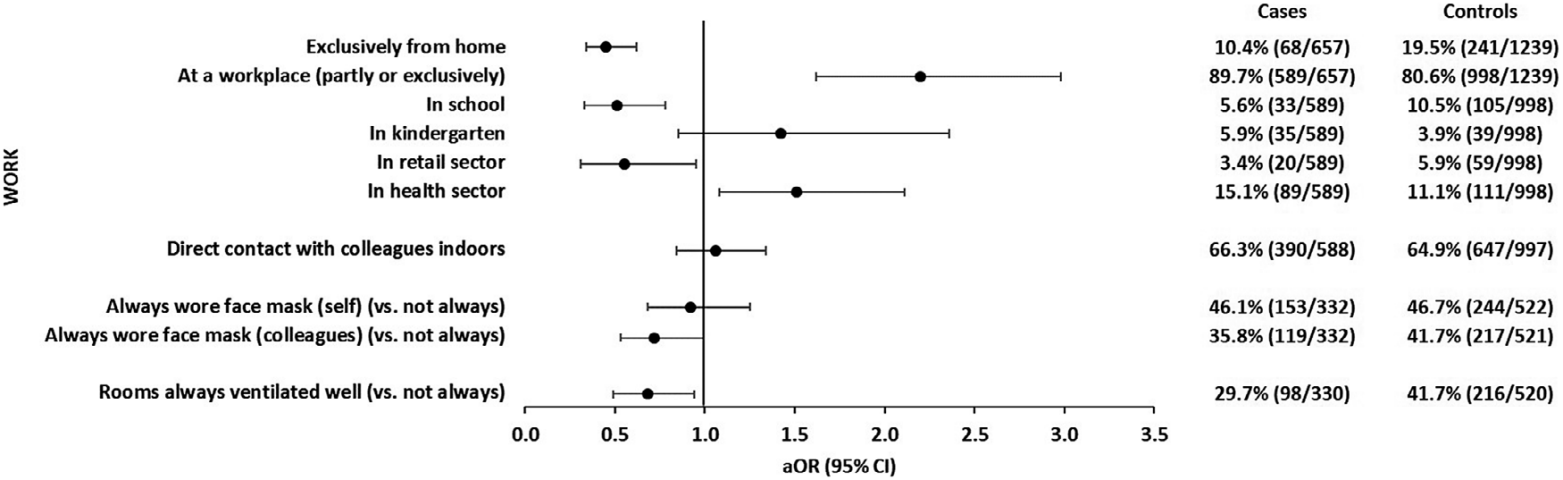
Adjusted odds ratios with 95% confidence intervals of workplace-related associations with symptomatic SARS-CoV-2 infections. Each exposure was analysed in a logistic regression model with five adjusting covariables (see the ‘Methods’ section for details). Analysis of the association with face masks when in direct contact with colleagues and with room ventilation was restricted to participants who reported direct contact indoors with at least one colleague (332 cases; 522 controls). Observations with missing values in the variable of interest or any of the five adjusting covariables were excluded from analysis.

Among participants who worked, those who worked exclusively from home were significantly less likely to have a symptomatic SARS-CoV-2 infection (aOR: 0.5; 95% CI: 0.3–0.6). Correspondingly, risk of symptomatic SARS-CoV-2 infection was increased for participants whose work was partly or exclusively conducted at a workplace other than their home (aOR: 2.2; 95% CI: 1.6–3.0). Work in a school including contact with children (aOR: 0.5; 95% CI: 0.3–0.8) and work in the retail sector including contact with customers (aOR: 0.6; 95% CI: 0.3–1.0) were negatively associated with symptomatic SARS-CoV-2 infections, whereas work in health care including contact with patients was positively associated (aOR: 1.5; 95% CI: 1.1–2.1). We did not observe a statistically significant association of symptomatic SARS-CoV-2 infections with work in a kindergarten (Figure 2 and Supplementary Table S2).

A negative association with symptomatic SARS-CoV-2 infections was found if participants reported that their colleagues had always worn face masks when in contact (indoors) with them (aOR: 0.7; 95% CI: 0.5–1.0), whereas there was no association if persons reported that they themselves had always worn a face mask when in direct contact with colleagues (aOR: 0.9; 95% CI: 0.7–1.3). If direct contacts with colleagues occurred in rooms that were always ventilated well (compared with ‘not always’), risk of symptomatic SARS-CoV-2 infection was decreased (aOR: 0.7; 95% CI: 0.5–0.9) (Figure 2 and Supplementary Table S2).

#### Private contacts

**Figure 3.**
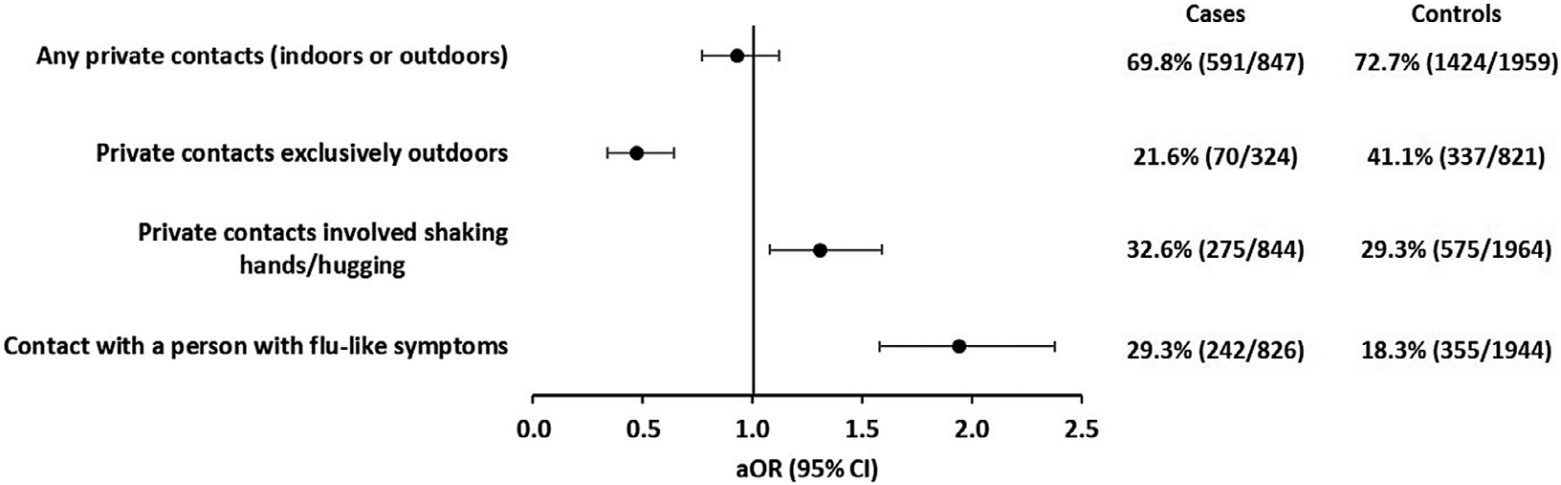
Adjusted odds ratios with 95% confidence intervals of private contacts-related associations with symptomatic SARS-CoV-2 infections. Each exposure (two categories: yes/no) was analysed in a logistic regression model with five adjusting covariables (see the ‘Methods’ section for details). Analysis of outdoor versus indoor contacts was restricted to participants who reported only indoor contacts or only outdoor contacts (324 case-patients; 821 controls). Observations with missing values in the variable of interest or any of the five adjusting covariables were excluded from analysis.

We defined direct private contacts as contacts for longer than 15 min at a distance of less than 1.5 m with persons not living in the same household. Direct private contacts exclusively outdoors were associated with a decreased risk of symptomatic SARS-CoV-2 infection (aOR: 0.5; 95% CI: 0.3–0.6) compared with private contacts exclusively indoors. If private contacts involved shaking hands or hugging, risk of symptomatic SARS-CoV-2 infection was increased (aOR: 1.3; 95% CI: 1.1–1.6). We observed a strong positive association with symptomatic SARS-CoV-2 infections if contact persons (at work or private contacts) had flu-like symptoms (aOR: 1.9; 95% CI: 1.6–2.4) (Figure 3 and Supplementary Table S3).

#### Social and other activities

**Figure 4.**
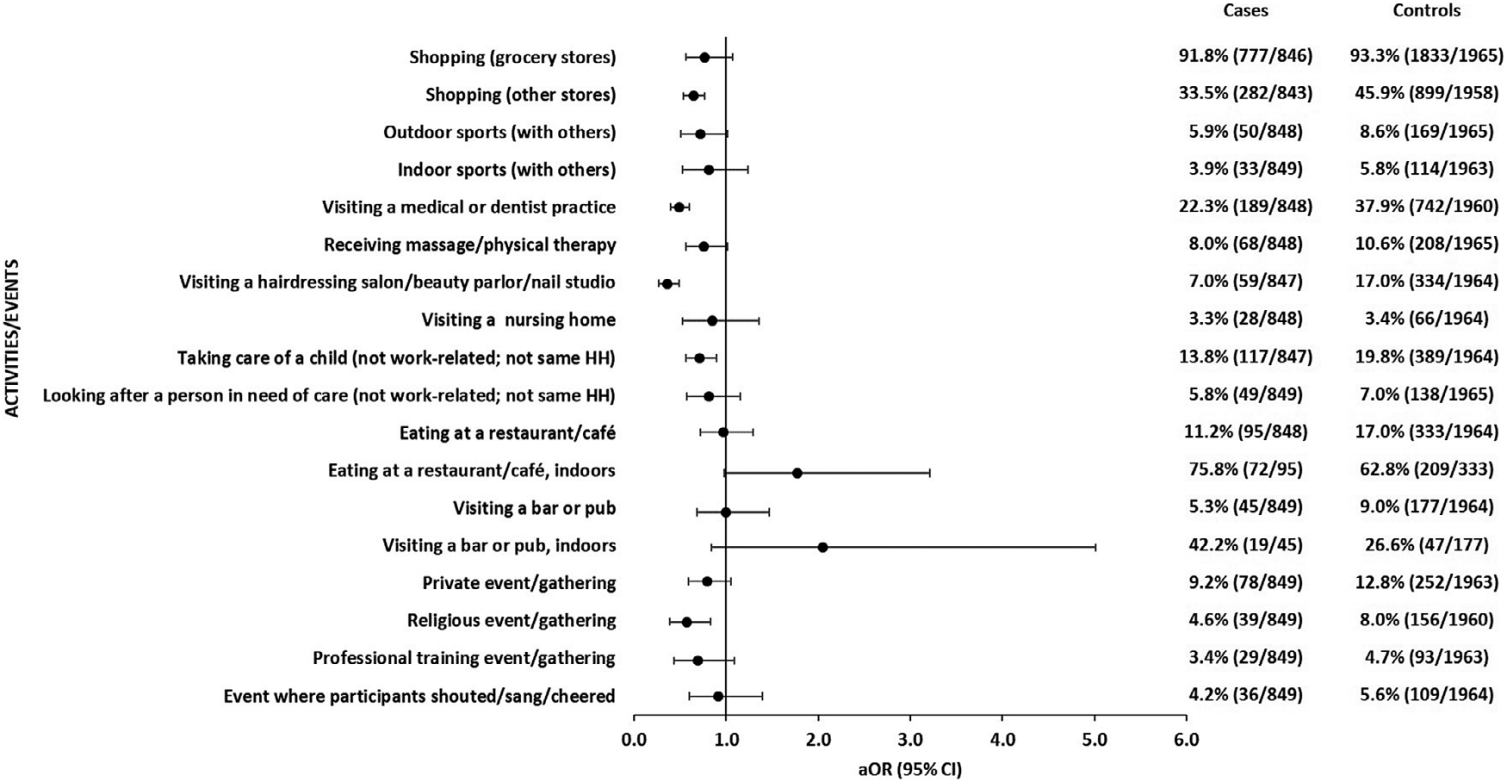
Adjusted odds ratios with 95% confidence intervals of associations of exposures (social and other activities) with symptomatic SARS-CoV-2 infections. Each single exposure was analysed in a logistic regression model with five adjusting covariables (see the ‘Methods’ section for details). Analysis of indoor eating was restricted to participants who reported eating at a restaurant (95 cases; 333 controls). Analysis of visiting a bar/pub indoors was restricted to participants who reported visiting a bar/pub (45 cases; 177 controls). Observations with missing values in the variable of interest or any of the five adjusting covariables were excluded from analysis. HH: household.

We analysed a variety of social activities that typically involve person-to-person contact. Shopping in grocery stores was not associated with symptomatic SARS-CoV-2 infections. Interestingly, more controls than case-patients reported shopping in non-grocery stores, which resulted in a negative association. Nearly every participant reported to have always worn a face mask when shopping in grocery or non-grocery stores. We also observed a negative association with visiting a medical or dentist practice, and with visiting a hairdressing salon, beauty parlour, or nail salon (Figure 4 and Supplementary Table S4).

Taking care of a child not living in the same household (but not professionally in a kindergarten or school), for example, as grandparents or neighbours, was negatively associated with symptomatic SARS-CoV-2 infections (Figure 4 and Supplementary Table S4). The proportion of participants who reported to have always worn a face mask when taking care of a child not living in the same household was small (<1% of case-patients; 4% of controls).

Eating at a restaurant or café, or visiting a bar or pub was not associated with an increased risk of symptomatic SARS-CoV-2 infection. We observed a tendency (not statistically significant) toward a positive association when we compared indoor restaurant or bar visits to outdoor visits. Participating in private events or gatherings, for example, birthday parties and weddings, was not associated with sporadic symptomatic SARS-CoV-2 infections. The proportion of controls who reported to have always worn a face mask when participating in private events was higher than the proportion of case-patients (13% vs. 6%). Interestingly, participating in a religious event (church service or similar) was associated with a decreased risk of symptomatic SARS-CoV-2 infection (Figure 4 and Supplementary Table S4). The proportion of those who reported to have always worn a face mask when visiting a religious event was high (90% of case-patients; 87% of control-persons). Public and private gatherings were highly restricted during long time periods of our study. Therefore, only about 23% of all study participants reported taking part in any such event (20% of cases; 29% of controls). We could not analyse a number of activities that we queried because the number of participants who reported them was too small (see Supplementary Table S4 for details).

#### Public transport and domestic travelling

**Figure 5.**
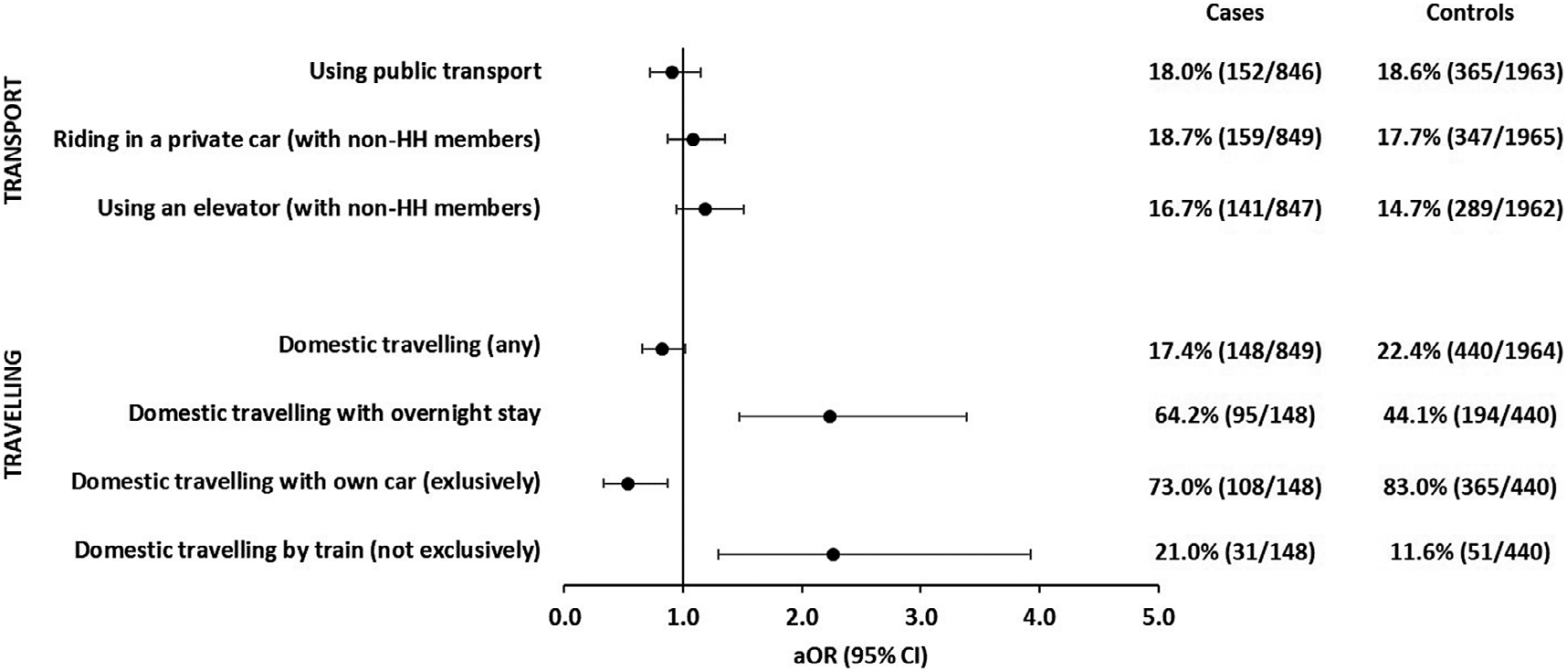
Adjusted odds ratios with 95% confidence intervals of associations of exposures related to transport and travelling with symptomatic SARS-CoV-2 infections. Each single exposure was analysed in a logistic regression model with five adjusting covariables (see the ‘Methods’ section for details). Analysis of details of travelling was restricted to participants who reported any domestic travelling (148 cases; 440 controls). Observations with missing values in the variable of interest or any of the five adjusting covariables were excluded from analysis. HH: household.

The use of local public transport was not associated with symptomatic SARS-CoV-2 infections. Almost all participants reported to have always worn a face mask when using local public transport (100% of case-patients; 99% of control-persons). Other means of transport (transport in a private car; using an elevator with persons not living in the same household) were not associated with symptomatic SARS-CoV-2 infections (Figure 5 and Supplementary Table S5).

Persons who reported travelling abroad had been excluded from study participation. Travelling within Germany was not associated with symptomatic SARS-CoV-2 infections. However, among those who reported domestic travelling, risk of sporadic SARS-CoV-2 infection was increased if travelling involved at least one overnight stay (aOR: 2.2; 95% CI: 1.5–3.4). Among domestic travellers, a negative association with symptomatic SARS-CoV-2 infections was observed if they exclusively used a private car (aOR: 0.5; 95% CI: 0.3–0.9). When travel was conducted by train (or a combination of trains with other means of transportation), risk of sporadic SARS-CoV-2 infection was increased (aOR: 2.3; 95% CI: 1.3–3.9) (Figure 5 and Supplementary Table S5).

#### Personal characteristics

**Figure 6.**
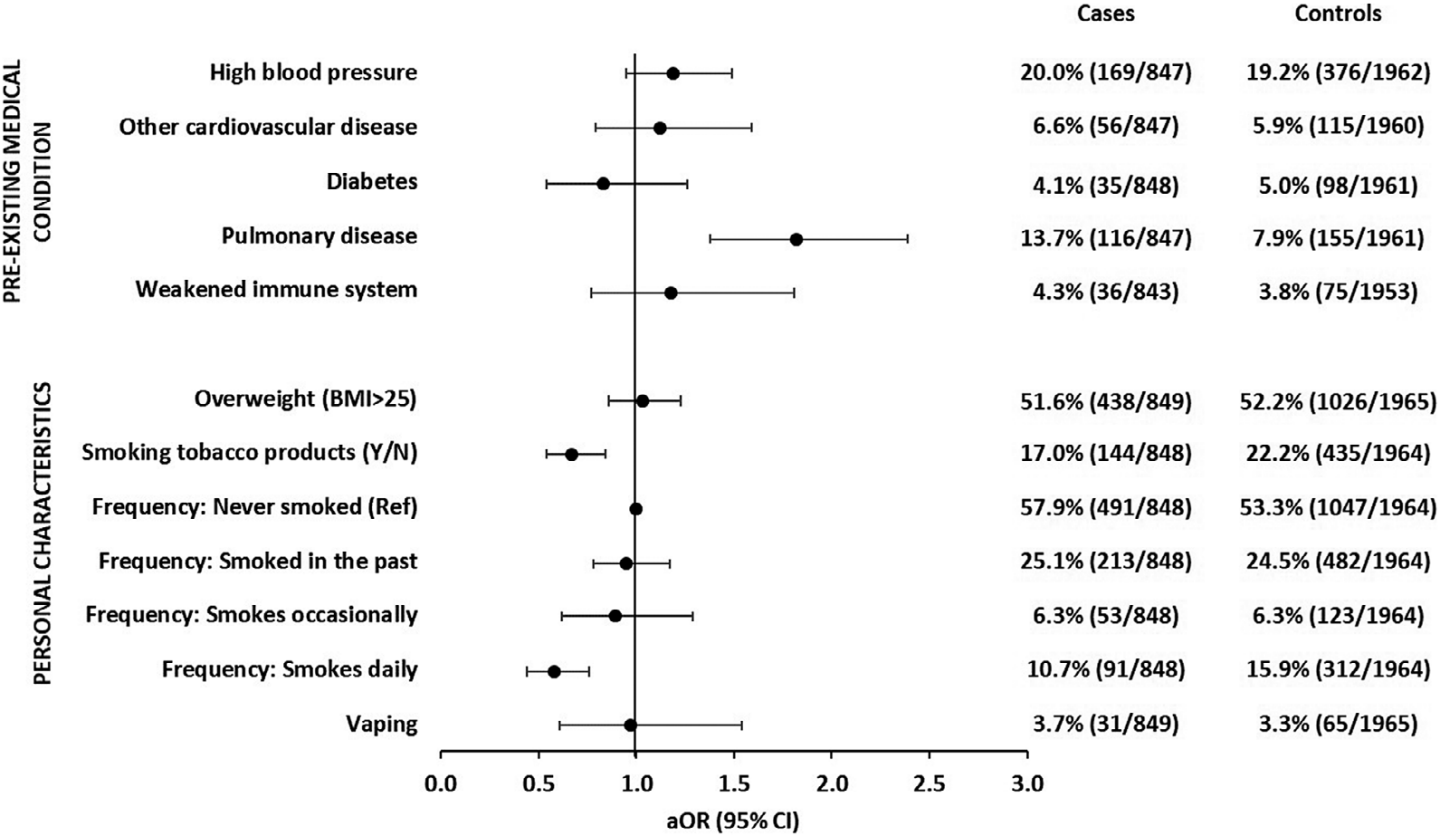
Adjusted odds ratios with 95% confidence intervals of associations of pre-existing medical conditions and personal characteristics with symptomatic SARS-CoV-2 infections. Each single variable was analysed in a logistic regression model with five adjusting covariables (see the ‘Methods’ section for details). The variable ‘frequency of smoking’ had four categories. Other variables shown in Figure 6 had two categories (yes/no). Observations with missing values in the variable of interest or any of the five adjusting covariables were excluded from analysis. BMI: body mass index; Ref: reference category; Y/N: yes versus no.

We analysed if certain pre-existing medical conditions, body mass index (BMI), or smoking behaviour were associated with symptomatic SARS-CoV-2 infections. Of the five medical conditions that we queried, only pre-existing pulmonary diseases, for example, asthma, chronic bronchitis, and chronic obstructive pulmonary disease, were associated with symptomatic SARS-CoV-2 infections (aOR: 1.8; 95% CI: 1.4–2.4) (Figure 6 and Supplementary Table S6). We did not observe an association with BMI. Daily smoking of tobacco products (cigarettes, cigars, pipe, and shisha) was statistically associated with a decreased risk of symptomatic SARS-CoV-2 infection (aOR: 0.6; 95% CI: 0.4–0.8) compared with smoking occasionally, having smoked in the past only, or having never smoked. Smoking was not correlated with pre-existing pulmonary disease in our study.

## Discussion

The CoViRiS study was planned and started in a relatively early phase of the COVID-19 pandemic when it was largely unknown which behavioural and societal activities might be associated with SARS-CoV-2 infections. Due to lack of specific evidence-based knowledge, NPIs were implemented with the general aim to reduce person-to-person contacts and thereby reduce SARS-CoV-2 transmissions, for example, by closing shops and restaurants and banning cultural events. The goal of our study was to identify behavioural and societal determinants associated with symptomatic SARS-CoV-2 infections. Several comparable case–control studies from other countries have been published in the meantime [8–15]. The focus of our study was on determinants of sporadic infections. We investigated exposures from a variety of contexts and settings, for which, at the time, associations with symptomatic SARS-CoV-2 infections could be hypothesized. Overall, we found few exposures positively associated with SARS-CoV-2 infections. This may indicate that the exposure-related NPIs in place during our study were useful in preventing SARS-CoV-2 infections in the settings that we investigated. The lack of observed associations may also indicate that SARS-CoV-2 infection risk was mainly driven by determinants not measured in our study. Other studies found that SARS-CoV-2 transmission in households played an important role [14, 16]. We excluded case-patients who were likely infected through household transmission because we were interested in determinants of sporadic SARS-CoV-2 infections in other settings.

One of the settings we focused on was the workplace. As a public health measure to reduce person-to-person contacts, enabling and conducting work from home was strongly encouraged and temporarily even mandated (where possible) during the pandemic in Germany. We found that the risk of symptomatic SARS-CoV-2 infections was indeed lower for persons who exclusively worked from home compared with people who partially or always worked at another workplace. Case–control studies in other countries provided similar findings [10, 12, 14, 15, 17]. Our study was inconclusive regarding the use of face masks at the workplace. Wearing a face mask when directly interacting with co-workers was not associated with SARS-CoV-2 infections in our study. However, we observed a negative association if co-workers always wore a face mask. Other studies demonstrated that face masks can be protective when in close contact with other people [18–21]. Work in the health sector with contact to patients was associated with an increased risk of symptomatic SARS-CoV-2 infection, even though we excluded health care personnel who knew that they had had contact with COVID-19 patients without adequate personal protective equipment from our study. Our results confirm those of others who also observed an increased COVID-19 risk for health care workers [12, 15, 22, 23]. We have no plausible explanation why working in a school, working in the retail sector, or privately taking care of a child (not living in the same household) were negatively associated with SARS-CoV-2 infection.

We also investigated possible exposures through close personal contacts that were not work-related (private contacts). In our study, we found that only indoor contacts were associated with an increased risk of symptomatic SARS-CoV-2 infections. Other studies also demonstrated that the risk of SARS-CoV-2 transmission was higher indoors than outdoors [24, 25]. Private contacts that involved shaking hands or hugging, and contact with a person with flu-like symptoms (presumably often due to undiagnosed COVID-19) were associated with an increased COVID-19 risk in our study. Recommendations to reduce close person-to-person contacts and to stay at home with flu-like symptoms are in accordance with these results.

We were interested in a variety of social activities, such as visiting museums or theatres, or participating in events with a large number of people, as possible exposures associated with SARS-CoV-2 infections. However, testing our hypotheses was impaired because many of these activities were either prohibited or only allowed with restrictions during our study. In consequence, only few participants engaged in some of the activities we were interested in and associations could not be analysed. Furthermore, some restricted activities were only possible for people with proof of vaccination, previous SARS-CoV-2 infection, or a recent negative SARS-CoV-2 antigen test, and often required mandatory use of face masks in addition. Of the activities we were able to analyse, only eating at restaurants and visiting bars indoors showed a tendency toward a positive association with sporadic SARS-CoV-2 infections, albeit not statistically significant. All other activities shown in Figure 4 and Supplementary Table S4 were not positively associated with SARS-CoV-2 infections. One reason may be that the power of our study to detect such associations became insufficient because only few participants reported these activities due to the restrictions. Another reason could be that the recommended or mandated health protection measures, for example, mandatory face mask, when engaging in these activities prevented SARS-CoV-2 transmissions. Comparable studies conducted in other countries (Denmark, the United States, France, and the United Kingdom) identified, for example, visiting restaurants or bars, visiting fitness centres/gyms, or attending professional or private gatherings as risk factors for SARS-CoV-2 infections [8–11, 13–15]. Health protection measures regarding, for example, restaurant visits, may have been less strict at the time when the studies were conducted in these countries compared to measures that were in place in Germany at the time of our study. This may be one possible explanation for the differing results.

Surprisingly, some activities, such as shopping in non-grocery stores, visiting a medical or dentist practice, visiting a hair dresser/cosmetics salon/nail studio, and visiting a religious service, were negatively associated with symptomatic SARS-CoV-2 infections in our study. Statistically significant negative associations with visiting shops/supermarkets/malls, attending health care appointments, and visiting hairdressers were also observed in case–control studies in other countries [8, 12, 15]. The authors speculated that mandatory health protection measures in combination with high compliance by study participants may contribute to the observed negative associations [12], or that the negative associations may be due to confounding by an unknown factor [15]. This may also be true for our study.

In our study, using local public transport was not associated with symptomatic SARS-CoV-2 infections, which confirms results from other comparable studies [8, 10, 12–15]. Compliance with mandatory use of face masks in public transport was reported by all case-patients and nearly all controls, which may explain this finding. Travelling within Germany was associated with an increased risk of symptomatic SARS-CoV-2 infections if it included at least one overnight stay away from home or the use of trains. Face masks became mandatory on long-distance trains early on in the pandemic, but could be temporarily removed for eating and drinking. Unfortunately, we did not ask participants if they wore face masks when travelling by train. Differences in compliance between case-patients and controls may have contributed to the observed association.

We investigated several personal characteristics including smoking behaviour as possible determinants of symptomatic SARS-CoV-2 infections. The negative association with daily smoking was unexpected, but has also been reported by others [26–31]. The seemingly protective effect of smoking is discussed controversially in the scientific literature [29, 32]. One biological explanation could be the reduction of angiotensin-converting enzyme 2 (ACE2) expression in the respiratory tract by certain compounds present in cigarette smoke, as has been shown for cell cultures [33]. However, contrary findings on the effect of smoking on ACE2 expression were also reported [34]. ACE2 is the receptor that binds SARS-CoV-2. Other studies have shown that smoking is associated with severe disease courses, including hospitalization, ICU treatment, and death due to COVID-19 [35, 36]. Therefore, smokers may have been less likely to be recruited as case-patients for our study. In contrast, smoking status would not have influenced the likelihood of study participation among the control group. This may have biased our results toward a negative association of smoking with symptomatic SARS-CoV-2 infections [37].

Some of our findings are difficult to interpret. Several of the unexpected negative associations were already discussed above. We are aware that our study has limitations. As in all observational studies, insufficient adjustment of our models for confounding may have skewed the observed associations of some of the queried activities with SARS-CoV-2 infections. We attempted to minimize biases and adjust for possible confounding factors by matching and including an adjustment set of variables in our logistic regression models. We took different temporary or regional restrictions of activities into account by recruiting controls from the same regions as case-patients. However, logistical and other constraints, including the requirement to obtain study consent in written form in addition to verbal consent, made it difficult to interview case-patients in a timely manner and hampered the optimal alignment of the queried 10-day time period of case-patients and controls. More controls than case-patients were interviewed in the time periods between COVID-19 pandemic waves, when some restrictive NPIs were lifted and certain activities were possible. Thus, controls may have had a greater chance to engage in these activities. We attempted an adjustment by including the local 7-day SARS-Cov-2 incidence into our regression models. The local 7-day incidence correlated with the stringency of regional NPIs. In our study, alignment of case and control interviews was better during the third pandemic wave in Germany. It was reassuring that our analyses with the restricted dataset did not yield substantially different results compared with the complete dataset.

Recall bias may have distorted the results of our case–control study. The time period that we queried the case-patients about (10-day period before onset of symptoms) dated further back than the time period that we queried the control-persons about (10-day period before the interview). Thus, case-patients may not have been able to recall certain activities as accurately as controls, which could result in an underestimation of the strength of an association. But case-patients may also recall certain exposures more accurately than controls, because they have thought about possible exposures more intensely. This could result in an overestimation of an association [38].

Observational studies, including case–control studies that are designed to identify risk factors and protective factors, are prone to biases and confounding. Therefore, any observed statistical association between an exposure and an outcome should be interpreted with caution and does not necessarily indicate a causal effect. This limits the usefulness of observational studies to assess the effectiveness of specific interventions such as NPIs. Experimental studies, for example, randomized controlled trials, or other study designs, would be better suited for the evaluation of the effectiveness of NPIs, but are not always possible to conduct, for example, due to ethical considerations [39, 40]. In context with other diligently performed studies, and if carefully analysed and cautiously interpreted, keeping all limitations in mind, observational studies may contribute to the overall body of evidence regarding the effectiveness of NPIs.

Despite the limitations of our study, our findings may be useful for putting into perspective the recommendations that were mainly aimed at reducing the number of person-to-person contacts during the COVID-19 pandemic. Our study results suggest that the risk of symptomatic SARS-CoV-2 infections can be reduced by work from home, when possible. Other recommendations whose usefulness is supported by our study results were: to wear face masks and ensure adequate ventilation indoors [41] when in direct contact with colleagues at work; to reduce personal contacts indoors and preferably meet outdoors; and to reduce person-to-person contacts when having flu-like symptoms by staying at home [4].

## Supporting information

Rosner et al. supplementary material Rosner et al. supplementary material

## Data Availability

Additional data are available as the Supplementary Material. Data will be made available upon reasonable request to the corresponding first or last author.

## Supplementary material

For supplementary material accompanying this paper visit http://doi.org/10.1017/S0950268824000050. click here to view supplementary material
