# Supplementary material for: Case–control study of behavioural and societal risk factors for sporadic SARS-CoV-2 infections, Germany, 2020–2021 (CoViRiS study)
Source: Epidemiol Infect. 2024 Jan 15;152:e16. doi: 10.1017/S0950268824000050 (PMC10894885; doi:10.1017/S0950268824000050)
Supplement: Rosner et al. supplementary material [file S0950268824000050sup001.docx]

**Case-Control Study of Behavioral and Societal Risk Factors** **for Sporadic SARS-CoV-2 Infections, Germany, 2020-2021 (CoViRiS Study)**

**Authors**

Bettina M Rosner^1*^, Gerhard Falkenhorst^1^, Isabella Kumpf^1^, Maren Enßle^1^, Andreas Hicketier^1^, Achim Dörre^1^, Klaus Stark^1^, Hendrik Wilking^1^

**Affiliations**

^1^Department of Infectious Disease Epidemiology, Robert Koch Institute, Berlin, Germany

**Supplementary Material**

**
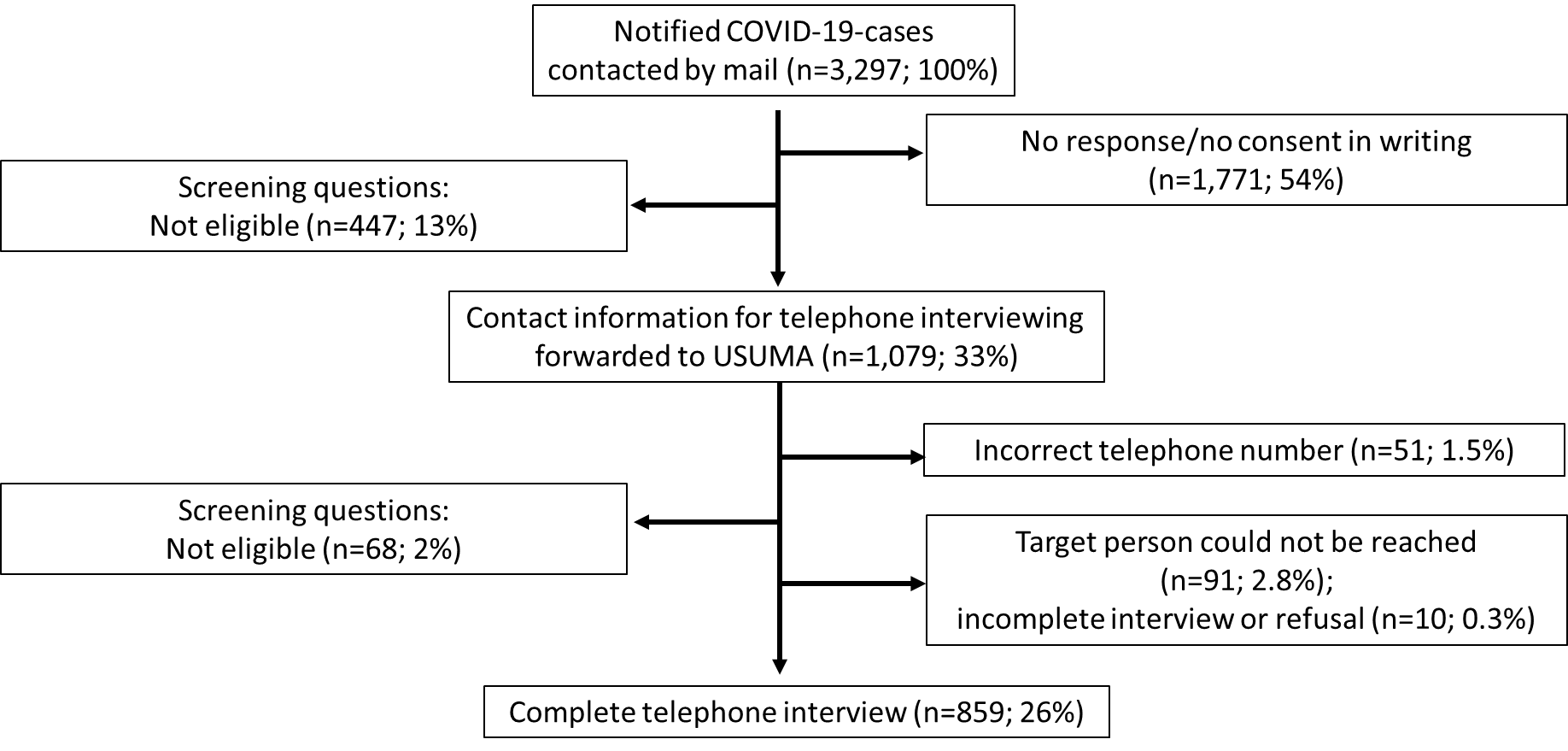
**

**Figure S1: Participation of COVID-19 case-persons in the study.**

**Table S1: Associations of household characteristics with symptomatic SARS-CoV-2 infections, case-control study, Germany, 2020-2021.** Each single exposure variable (household size; child in household; household with risk group) was analysed in a logistic regression model with 5 adjusting covariables (age group, sex, urban/rural type of district of residence, vaccination status and 7-day-incidence). Observations with missing values in the variable of interest or any of the 5 adjusting covariables were excluded from analysis.

| **Variable** | **Cases**  **% (n/N)** | **Controls**  **% (n/N)** | **aOR^a^** | **95%-CI^b^** | ***P*-value** |
| --- | --- | --- | --- | --- | --- |
| **Household size** |  |  |  |  |  |
| 1 person | 16.1% (137/849) | 17.1% (335/1965) | Reference |  |  |
| 2–4 persons | 77.9% (661/849) | 74.5% (1463/1965) | 1.02 | 0.80–1.28 | 0.895 |
| More than 4 persons | 6.0% (51/849) | 8.5% (167/1965) | 0.69 | 0.46–1.03 | 0.070 |
| **Household with child**  **<18 years of age^c^** | 36.4% (259/712) | 42.2% (687/1629) | 0.76 | 0.60–0.95 | 0.017 |
| Child <6 years of age^d^ | 40.5% (105/259) | 34.1% (234/687) | 1.55 | 1.11–2.16 | 0.009 |
| Child 6–10 years of age^d^ | 38.6% (100/259) | 38.9% (267/687) | 1.00 | 0.72–1.38 | 0.988 |
| Child 11–17 years of age^d^ | 49.4% (128/259) | 58.8% (404/687) | 0.57 | 0.41–0.79 | 0.001 |
| **Household with person of risk group^c,e^** | 25.3% (179/708) | 29.1% (472/1623) | 0.78 | 0.63–0.97 | 0.024 |

^a^adjusted odds ratio

^b^95%-confidence interval

^c^ if household size >1 person

^d^ in households with children: at least one child in this age group

^e^ possible risk groups, e.g., persons >60 years of age or/and with chronic diseases

**Table S2: Associations of workplace characteristics with symptomatic SARS-CoV-2 infections, case-control study, Germany, 2020**–**2021.** Each single exposure variable was analysed in a logistic regression model with 5 adjusting covariables (age group, sex, urban/rural type of district of residence, vaccination status and 7-day-incidence). Observations with missing values in the variable of interest or any of the 5 adjusting covariables were excluded from analysis. Other settings of work could not be analysed because the numbers in the strata were too small (<3%): work in a nursing home; work in a hair/cosmetics/nail salon; work as bus or taxi driver; work in the gastronomy sector; work in a slaughterhouse.

| **Variable** | **Cases**  **% (n/N)** | **Controls**  **% (n/N)** | **aOR^a^** | **95%-CI^b^** | ***P*-value** |
| --- | --- | --- | --- | --- | --- |
| **Work exclusively from home** | 10.4% (68/657) | 19.5% (241/1239) | 0.45 | 0.34–0.62 | <0.001 |
| **Work partly or exclusively at a workplace other than home** | 89.7% (589/657) | 80.6% (998/1239) | 2.20 | 1.62–2.98 | <0.001 |
| **Setting of work** |  |  |  |  |  |
| School^c,d^ | 5.6% (33/589) | 10.5% (105/998) | 0.51 | 0.33–0.78 | 0.002 |
| Kindergarten^c,d^ | 5.9% (35/589) | 3.9% (39/998) | 1.42 | 0.85–2.36 | 0.180 |
| Retail sector^c,e^ | 3.4% (20/589) | 5.9% (59/998) | 0.55 | 0.31–0.95 | 0.032 |
| Health sector^c,f^ | 15.1% (89/589) | 11.1% (111/998) | 1.51 | 1.08–2.11 | 0.014 |
| **Direct contact^c,g^ with colleagues indoors (partly or exclusively)** | 66.3% (390/588) | 64.9% (647/997) | 1.06 | 0.84–1.34 | 0.614 |
| **Always^h^ wore face mask when in direct contact with colleagues^c,g,i^ (self)** | 46.1% (153/332) | 46.7% (244/522) | 0.92 | 0.68–1.25 | 0.594 |
| **Colleagues always^h^ wore face masks when in direct contact^c,g,i^ (with self)** | 35.8% (119/332) | 41.7% (217/521) | 0.72 | 0.53–0.99 | 0.041 |
| **Rooms always^h^ ventilated well when in direct contact with colleagues^c,g,i^** | 29.7% (98/330) | 41.7% (216/520) | 0.68 | 0.49–0.94 | 0.021 |

^a^adjusted odds ratio

^b^95%-confidence interval

^c^ work partly or exclusively at a workplace other than home

^d^ includes contact with children

^e^ includes contact with customers

^f^ includes contact with patients

^g^ direct contact defined as distance <1.5 meters

^h^ versus “not always”

^i^ direct contact indoors for >15 minutes

**Table S3: Associations of private contacts with symptomatic SARS-CoV-2 infections, case-control study, Germany, 2020**–**2021.** Each single exposure variable was analysed in a logistic regression model with 5 adjusting covariables (age group, sex, urban/rural type of district of residence, vaccination status and 7-day-incidence). Observations with missing values in the variable of interest or any of the 5 adjusting covariables were excluded from analysis. Direct private contacts were defined as contacts for >15 minutes at a distance of <1.5 meters with persons not living in the same household.

| **Variable** | **Cases**  **% (n/N)** | **Controls**  **% (n/N)** | **aOR^a^** | **95%-CI^b^** | ***P*-value** |
| --- | --- | --- | --- | --- | --- |
| **Any direct private contacts (indoors or outdoors)** | 69.8% (591/847) | 72.7% (1424/1959) | 0.93 | 0.77–1.12 | 0.439 |
| **Direct private contacts exclusively outdoors^c^** | 21.6 % (70/324) | 41.1% (337/829) | 0.47 | 0.34-0.64 | <0.001 |
| **Direct private contacts involved shaking hands, hugging** | 32.6% (275/844) | 29.3% (575/1964) | 1.31 | 1.08–1.59 | 0.007 |
| **Any contact with a person with flu-like symptoms^d^** | 29.3% (242/826) | 18.3% (355/1944) | 1.94 | 1.58–2.38 | <0,001 |

^a^adjusted odds ratio

^b^95%-confidence interval

^c^versus private contacts exclusively indoors

^d^includes private or work contacts for less than 15 minutes

**Table S4: Associations of activities that may involve person-to-person contacts with symptomatic SARS-CoV-2 infections, case-control study, Germany, 2020-2021.** Each single exposure variable was analysed in a logistic regression model with 5 adjusting covariables (age group, sex, urban/rural type of district of residence, vaccination status and 7-day-incidence). Observations with missing values in the variable of interest or any of the 5 adjusting covariables were excluded from analysis. Other activities could not be analysed because the numbers in strata were too small (<3%): visiting a gaming arcade, betting office or similar location; visiting a fitness studio/gym; visiting a public sauna; visiting patients in hospitals; visiting museums; visiting theatres; participating in choir rehearsals or choir performances; participating in rehearsals or performances of orchestras with wind instruments; participating in rehearsals or performances of theater groups; visiting street fairs or carnival events; visiting sports events; participating in demonstrations or rallies; visiting music concerts; participating in company staff meetings, meetings of associations or similar meetings; visiting conferences or trade fairs.

| **Variable** | **Cases**  **% (n/N)** | **Controls**  **% (n/N)** | **aOR^a^** | **95%-CI^b^** | ***P*-value** |
| --- | --- | --- | --- | --- | --- |
| **Shopping in grocery stores** | 91.8% (777/846) | 93.3% (1833/1965) | 0.77 | 0.56–1.07 | 0.121 |
| **Shopping in other stores** | 33.5% (282/843) | 45.9% (899/1958) | 0.65 | 0.54–0.77 | <0.001 |
| **Outdoor sports in club/team (with others)** | 5.9% (50/848) | 8.6% (169/1965) | 0.72 | 0.51–1.02 | 0.066 |
| **Indoor sports in club/team (with others)** | 3.9% (33/849) | 5.8% (114/1963) | 0.81 | 0.53–1.24 | 0.331 |
| **Visiting a medical or dentist practice** | 22.3% (189/848) | 37.9% (742/1960) | 0.49 | 0.40–0.60 | <0.001 |
| **Receiving massage or physical therapy or similar** | 8.0% (68/848) | 10.6% (208/1965) | 0.76 | 0.56–1.02 | 0.071 |
| **Visiting hairdressing salon, beauty parlor, or nail studio** | 7.0% (59/847) | 17.0% (334/1964) | 0.36 | 0.27–0.49 | <0.001 |
| **Visiting someone in nursing home** | 3.3% (28/848) | 3.4% (66/1964) | 0.85 | 0.53–1.36 | 0.494 |
| **Privately taking care of a child (not living in the same household)** | 13.8% (117/847) | 19.8% (389/1964) | 0.71 | 0.56–0.90 | 0.004 |
| **Privately looking after a person in need of care (not living in the same household)** | 5.8% (49/849) | 7.0% (138/1965) | 0.81 | 0.57–1.15 | 0.237 |
| **Eating in a restaurant/café** | 11.2% (95/848) | 17.0% (333/1964) | 0.97 | 0.72–1.29 | 0.812 |
| Eating in a restaurant/café, indoors at least once | 75.8% (72/95) | 62.8% (209/333) | 1.77 | 0.98–3.21 | 0.059 |
| **Visiting a bar or pub** | 5.3% (45/849) | 9.0% (177/1964) | 1.00 | 0.68–1.47 | 0.998 |
| Visiting a bar or pub, indoors at least once | 42.2% (19/45) | 26.6% (47/177) | 2.05 | 0.84–5.01 | 0.114 |
| **Participating in a private event (e.g. birthday party, wedding, funeral)** | 9.2% (78/849) | 12.8% (252/1963) | 0.79 | 0.59–1.05 | 0.110 |
| **Participating in a religious event (e.g. church service)** | 4.6% (39/849) | 8.0% (156/1,960) | 0.57 | 0.39–0.83 | 0.004 |
| **Participating in professional training events, university seminars, or events at adult education centers** | 3.4% (29/849) | 4.7% (93/1963) | 0.69 | 0.43–1.09 | 0.112 |
| **Visiting an event where participants shouted, sang or cheered** | 4.2% (36/849) | 5.6% (109/1964) | 0.91 | 0.60–1.39 | 0.668 |
| **Participating in any event^c^** | 20.3% (172/849) | 28.5% (559/1961) | 0.69 | 0.56–0.86 | 0.001 |

^a^adjusted odds ratio

^b^95%-confidence interval

^c^Any of the queried events: private party/event; public event, e.g., carnival, street party; demonstration/rally or similar; sports event; religious event; adult educational/training event; workplace event; cultural event (musical event; theater; movie theater); professional conference or similar

**Table S5: Associations of public transportation or domestic traveling with symptomatic SARS-CoV-2 infections, case-control study, Germany, 2020-2021.** Each single exposure variable was analysed in a logistic regression model with 5 adjusting covariables (age group, sex, urban/rural type of district of residence, vaccination status and 7-day-incidence). Observations with missing values in the variable of interest or any of the 5 adjusting covariables were excluded from analysis. The following variables were not analysed because the numbers in strata were too small: transport by taxi/other service; travel by bus/coach; travel by boat/ship; travel by plane; travel by bicycle or motorcycle; location of overnight stay during travel (private home; hotel; vacation rental apartment; campground or similar).

| **Variable** | **Cases**  **% (n/N)** | **Controls**  **% (n/N)** | **aOR^a^** | **95%-CI^b^** | ***P*-value** |
| --- | --- | --- | --- | --- | --- |
| **Using public transportation** | 18.0% (152/846) | 18.6% (365/1963) | 0.91 | 0.72–1.15 | 0.441 |
| **Riding in a private car (with non-household members)** | 18.7% (159/849) | 17.7% (347/1965) | 1.08 | 0.87–1.35 | 0.487 |
| **Using an elevator (with non-household members)** | 16.7% (141/847) | 14.7% (289/1962) | 1.19 | 0.94–1.51 | 0.153 |
| **Domestic traveling** | 17.4% (148/849) | 22.4% (440/1964) | 0.82 | 0.66–1.02 | 0.076 |
| Domestic traveling with at least one overnight stay | 64.2% (95/148) | 44.1% (194/440) | 2.23 | 1.47–3.39 | <0.001 |
| Travel exclusively with own car | 73.0% (108/148) | 83.0% (365/440) | 0.54 | 0.33–0.87 | 0.013 |
| Travel by train (not exclusively) | 21.0% (31/148) | 11.6% (51/440) | 2.26 | 1.30–3.92 | 0.004 |

^a^adjusted odds ratio

^b^95%-confidence interval

**Table S6: Associations of personal characteristics with symptomatic SARS-CoV-2 infections, case-control study, Germany, 2020-2021.** Each single exposure variable was analysed in a logistic regression model with 5 adjusting covariables (age group, sex, urban/rural type of district of residence, vaccination status and 7-day-incidence). Observations with missing values in the variable of interest or any of the 5 adjusting covariables were excluded from analysis.

| **Variable** | **Cases**  **% (n/N)** | **Controls**  **% (n/N)** | **aOR^a^** | **95%-CI^b^** | ***P*-value** |
| --- | --- | --- | --- | --- | --- |
| **Pre-existing medical condition** |  |  |  |  |  |
| High blood pressure | 20.0% (169/847) | 19.2% (376/1962) | 1.19 | 0.95–1.49 | 0.139 |
| Other cardiovascular disease | 6.6% (56/847) | 5.9% (115/1960) | 1.12 | 0.79–1.59 | 0.541 |
| Diabetes | 4.1% (35/848) | 5.0% (98/1961) | 0.83 | 0.54–1.26 | 0.370 |
| Pulmonary disease, e.g., asthma, chronic bronchitis, COPD, lung emphysema) | 13.7% (116/847) | 7.9% (155/1961) | 1.82 | 1.38–2.39 | <0.001 |
| Weakened immune system (due to disease or medication) | 4.3% (36/843) | 3.8% (75/1953) | 1.18 | 0.77–1.81 | 0.455 |
| **Overweight (BMI>25)^c^** | 51.6% (438/849) | 52.2% (1026/1965 | 1.03 | 0.86–1.23 | 0.732 |
| **Smoking tobacco products^d^** | 17.0% (144/848) | 22.2% (435/1964) | 0.67 | 0.54–0.84 | <0.001 |
| **Frequency of smoking tobacco products** |  |  |  |  |  |
| Smokes daily | 10.7% (91/848) | 15.9% (312/1964) | 0.58 | 0.44–0.76 | <0.001 |
| Smokes occasionally | 6.3% (53/848) | 6.3% (123/1964) | 0.89 | 0.62–1.29 | 0.551 |
| Smoked in the past | 25.1% (213/848) | 24.5% (482/1964) | 0.95 | 0.78–1.17 | 0.653 |
| Never smoked | 57.9% (491/848) | 53.3% (1047/1964) | Reference |  |  |
| **Vaping (e-cigarettes or similar)** | 3.7% (31/849) | 3.3% (65/1965) | 0.97 | 0.61–1.54 | 0.897 |

^a^adjusted odds ratio (model with variables age group, sex, urban/rural type of residency, vaccination status and 7-day-incidence)

^b^95%-confidence interval

^c^versus BMI ≤25

^d^smokes daily or occasionally vs. smoked in the past or never smoked

**Table S7: Associations of household characteristics with symptomatic SARS-CoV-2 infections, case-control study, Germany, 2020-2021. Dataset limited to COVID-19 wave 3 in Germany (see Reference 7) (325 cases, 1083 controls).** Each single exposure variable (household size; child in household; household with risk group) was analysed in a logistic regression model with 5 adjusting covariables (age group, sex, urban/rural type of district of residence, vaccination status and 7-day-incidence). Observations with missing values in the variable of interest or any of the 5 adjusting covariables were excluded from analysis.

| **Variable** | **Cases**  **% (n/N)** | **Controls**  **% (n/N)** | **aOR^a^** | **95%-CI^b^** | ***P*-value** |
| --- | --- | --- | --- | --- | --- |
| **Household size** |  |  |  |  |  |
| 1 person | 16.5% (53/322) | 16.7% (181/1081) | Reference |  |  |
| 2–4 persons | 76.4% (246/322) | 75.4% (815/1081) | 0.91 | 0.64–1.29 | 0.584 |
| More than 4 persons | 7.1% (23/322) | 7.9% (85/1081) | 0.72 | 0.40–1.29 | 0.271 |
| **Household with child**  **<18 years of age^c^** | 34.2% (92/269) | 37.9% (341/899) | 0.71 | 0.50–1.01 | 0.054 |
| Child <6 years of age^d^ | 40.2% (37/92) | 26.4% (90/341) | 2.07 | 1.22–3.51 | 0.007 |
| Child 6–10 years of age^d^ | 44.6% (41/92) | 38.1% (130/341) | 1.05 | 0.63–1.75 | 0.844 |
| Child 11–17 years of age^d^ | 46.7% (43/92) | 66.9% (228/341) | 0.42 | 0.25–0.69 | 0.001 |
| **Household with person of risk group^c,e^** | 23.6% (63/267) | 31.4% (282/897) | 0.67 | 0.48–0.92 | 0.015 |

^a^adjusted odds ratio

^b^95%-confidence interval

^c^ if household size >1 person

^d^ in households with children: at least one child in this age group

^e^ possible risk groups, e.g., persons >60 years of age or/and with chronic diseases

**Table S8: Associations of workplace characteristics with symptomatic SARS-CoV-2 infections, case-control study, Germany, 2020**–**2021. Dataset limited to COVID-19 wave 3 in Germany (see Reference 7) (325 cases, 1083 controls).** Each single exposure variable was analysed in a logistic regression model with 5 adjusting covariables (age group, sex, urban/rural type of district of residence, vaccination status and 7-day-incidence). Observations with missing values in the variable of interest or any of the 5 adjusting covariables were excluded from analysis. Other settings of work could not be analysed because the numbers in the strata were too small (<3%): work in a nursing home; work in a hair/cosmetics/nail salon; work as bus or taxi driver; work in the gastronomy sector; work in a slaughterhouse.

| **Variable** | **Cases**  **% (n/N)** | **Controls**  **% (n/N)** | **aOR^a^** | **95%-CI^b^** | ***P*-value** |
| --- | --- | --- | --- | --- | --- |
| **Work exclusively from home** | 9.1% (22/243) | 22.2% (144/650) | 0.33 | 0.20-0.53 | <0.001 |
| **Work partly or exclusively at a workplace other than home** | 91.0% (221/243) | 77.9% (506/650) | 3.06 | 1.88-5.00 | <0.001 |
| **Setting of work** |  |  |  |  |  |
| School^c,d^ | 5.0% (11/221) | 10.7% (54/506) | 0.47 | 0.23–0.93 | 0.031 |
| Kindergarten^c,d^ | 8.1% (18/221) | 4.6% (23/506) | 2.03 | 1.03–4.01 | 0.042 |
| Retail sector^c,e^ | 2.7% (6/221) | 4.2% (21/506) | 0.58 | 0.23–1.48 | 0.254 |
| Health sector^c,f^ | 7.7% (17/221) | 11.5% (58/506) | 0.79 | 0.43–1.45 | 0.449 |
| **Direct contact^c,g^ with colleagues indoors (partly or exclusively)** | 62.4% (138/221) | 61.2% (309/505) | 1.03 | 0.74–1.44 | 0.857 |
| **Always^h^ wore face mask when in direct contact with colleagues^c,g,i^ (self)** | 48.7% (56/115) | 56.4% (137/243) | 0.74 | 0.46–1.19 | 0.218 |
| **Colleagues always^h^ wore face masks when in direct contact^c,g,i^ (with self)** | 35.7% (41/115) | 50.6% (123/243) | 0.55 | 0.33–0.89 | 0.015 |
| **Rooms always^h^ ventilated well when in direct contact with colleagues^c,g,i^** | 28.1% (32/114) | 39.0% (94/241) | 0.68 | 0.41–1.13 | 0.140 |

^a^adjusted odds ratio

^b^95%-confidence interval

^c^ work partly or exclusively at a workplace other than home

^d^ includes contact with children

^e^ includes contact with customers

^f^ includes contact with patients

^g^ direct contact defined as distance <1.5 meters

^h^ versus “not always”

^i^ direct contact indoors for >15 minutes

**Table S9: Associations of private contacts with symptomatic SARS-CoV-2 infections, case-control study, Germany, 2020**–**2021. Dataset limited to COVID-19 wave 3 in Germany (see Reference 7) (325 cases, 1083 controls).** Each single exposure variable was analysed in a logistic regression model with 5 adjusting covariables (age group, sex, urban/rural type of district of residence, vaccination status and 7-day-incidence). Observations with missing values in the variable of interest or any of the 5 adjusting covariables were excluded from analysis. Direct private contacts were defined as contacts for >15 minutes at a distance of <1.5 meters with persons not living in the same household.

| **Variable** | **Cases**  **% (n/N)** | **Controls**  **% (n/N)** | **aOR^a^** | **95%-CI^b^** | ***P*-value** |
| --- | --- | --- | --- | --- | --- |
| **Any direct private contacts (indoors or outdoors)** | 68.3% (220/322) | 66.8% (722/1081) | 1.03 | 0.78–1.36 | 0.826 |
| **Direct private contacts exclusively outdoors^c^** | 25.4% (33/130) | 37.5% (170/453) | 0.59 | 0.38-0.94 | 0.024 |
| **Direct private contacts involved shaking hands, hugging** | 25.5% (82/322) | 21.7% (235/1081) | 1.13 | 0.83–1.53 | 0.442 |
| **Any contact with a person with flu-like symptoms^d^** | 23.7% (75/317) | 12.6% (135/1073) | 2.22 | 1.60–3.08 | <0,001 |

^a^adjusted odds ratio

^b^95%-confidence interval

^c^versus private contacts exclusively indoors

^d^includes private or work contacts for less than 15 minutes

**Table S10: Associations of activities that may involve person-to-person contacts with symptomatic SARS-CoV-2 infections, case-control study, Germany, 2020-2021. Dataset limited to COVID-19 wave 3 in Germany (see Reference 7) (325 cases, 1083 controls).** Each single exposure variable was analysed in a logistic regression model with 5 adjusting covariables (age group, sex, urban/rural type of district of residence, vaccination status and 7-day-incidence). Observations with missing values in the variable of interest or any of the 5 adjusting covariables were excluded from analysis. Other activities could not be analysed because the numbers in strata were too small (<3%): visiting a gaming arcade, betting office or similar location; visiting a fitness studio/gym; visiting a public sauna; visiting patients in hospitals; visiting museums; visiting theatres; participating in choir rehearsals or choir performances; participating in rehearsals or performances of orchestras with wind instruments; participating in rehearsals or performances of theater groups; visiting street fairs or carnival events; visiting sports events; participating in demonstrations or rallies; visiting music concerts; participating in company staff meetings, meetings of associations or similar meetings; visiting conferences or trade fairs.

| **Variable** | **Cases**  **% (n/N)** | **Controls**  **% (n/N)** | **aOR^a^** | **95%-CI^b^** | ***P*-value** |
| --- | --- | --- | --- | --- | --- |
| **Shopping in grocery stores** | 93.5% (301/322) | 93.1% (1006/1081) | 1.08 | 0.65–1.82 | 0.760 |
| **Shopping in other stores** | 31.6% (101/320) | 38.8% (419/1079) | 0.74 | 0.57–0.97 | 0.031 |
| **Outdoor sports in club/team (with others)** | 8.1% (26/322) | 6.9% (74/1081) | 1.13 | 0.69–1.82 | 0.630 |
| **Indoor sports in club/team (with others)** | 2.5% (8/322) | 2.5% (27/1081) | 1.01 | 0.44–2.29 | 0.985 |
| **Visiting a medical or dentist practice** | 23.6% (76/322) | 37.0% (399/1079) | 0.57 | 0.42–0.76 | <0.001 |
| **Receiving massage or physical therapy or similar** | 8.1% (26/322) | 11.2% (121/1081) | 0.75 | 0.47–1.17 | 0.203 |
| **Visiting hairdressing salon, beauty parlor, or nail studio** | 8.7% (28/322) | 17.7% (191/1080) | 0.44 | 0.29–0.68 | <0.001 |
| **Visiting someone in nursing home** | 3.4% (11/321) | 3.2% (35/1081) | 1.00 | 0.50–2.03 | 0.995 |
| **Privately taking care of a child (not living in the same household)** | 15.9% (51/321) | 18.8% (203/1080) | 0.87 | 0.62–1.22 | 0.421 |
| **Privately looking after a person in need of care (not living in the same household)** | 4.0% (13/322) | 6.9% (75/1081) | 0.49 | 0.23–1.03 | 0.061 |
| **Eating in a restaurant/café** | 1.9% (6/321) | 4.0% (43/1081) | 0.69 | 0.27–1.78 | 0.448 |
| Eating in a restaurant/café, indoors at least once | 50.0%  (3/6) | 37.2% (16/43) | Not determined |  |  |
| **Visiting a bar or pub** | 0.9% (3/322) | 2.3% (25/1080) | 0.65 | 0.18–2.36 | 0.516 |
| Visiting a bar or pub, indoors at least once | 66.7% (2/3) | 16.0% (4/25) | Not determined |  |  |
| **Participating in a private event (e.g. birthday party, wedding, funeral)** | 5.0% (16/322) | 8.8% (95/1079) | 0.54 | 0.31–0.94 | 0.030 |
| **Participating in a religious event (e.g. church service)** | 3.1% (10/322) | 6.3% (68/1079) | 0.50 | 0.25–0.99 | 0.047 |
| **Participating in professional training events, university seminars, or events at adult education centers** | 3.1% (10/322) | 3.1% (33/1080) | 0.80 | 0.37–1.70 | 0.548 |
| **Visiting an event where participants shouted, sang or cheered** | 0.6% (2/322) | 1.4% (15/1080) | Not determined |  |  |
| **Participating in any event^c^** | 12.7% (41/322) | 19.9% (215/1079) | 0.56 | 0.39–0.82 | 0.002 |

^a^adjusted odds ratio

^b^95%-confidence interval

^c^Any of the queried events: private party/event; public event, e.g., carnival, street party; demonstration/rally or similar; sports event; religious event; adult educational/training event; workplace event; cultural event (musical event; theater; movie theater); professional conference or similar

**Table S11: Associations of public transportation or domestic traveling with symptomatic SARS-CoV-2 infections, case-control study, Germany, 2020-2021. Data set limited to COVID-19 wave 3 in Germany (see Reference 7) (325 cases, 1083 controls).** Each single exposure variable was analysed in a logistic regression model with 5 adjusting covariables (age group, sex, urban/rural type of district of residence, vaccination status and 7-day-incidence). Observations with missing values in the variable of interest or any of the 5 adjusting covariables were excluded from analysis. The following variables were not analysed because the numbers in strata were too small: transport by taxi/other service; travel by bus/coach; travel by boat/ship; travel by plane; travel by bicycle or motorcycle; location of overnight stay during travel (private home; hotel; vacation rental apartment; campground or similar).

| **Variable** | **Cases**  **% (n/N)** | **Controls**  **% (n/N)** | **aOR^a^** | **95%-CI^b^** | ***P*-value** |
| --- | --- | --- | --- | --- | --- |
| **Using public transportation** | 13.8% (44/319) | 18.4% (199/1080) | 0.64 | 0.44–0.95 | 0.026 |
| **Riding in a private car (with non-household members)** | 15.5% (50/322) | 15.5% (168/1081) | 0.91 | 0.63–1.30 | 0.586 |
| **Using an elevator (with non-household members)** | 16.6% (53/320) | 11.5% (124/1080) | 1.52 | 1.05–2.19 | 0.026 |
| **Domestic traveling** | 15.2% (49/322) | 17.2% (186/1080) | 0.92 | 0.64–1.31 | 0.625 |
| Domestic traveling with at least one overnight stay | 59.2% (29/49) | 42.5% (79/186) | 1.87 | 0.92–3.82 | 0.085 |
| Travel exclusively with own car | 79.6% (39/49) | 84.4% (157/186) | 0.73 | 0.30–1.77 | 0.482 |
| Travel by train (not exclusively) | 18.4% (9/49) | 9.7% (18/186) | 1.90 | 0.72–4.98 | 0.194 |

^a^adjusted odds ratio

^b^95%-confidence interval

**Table S12: Associations of personal characteristics with symptomatic SARS-CoV-2 infections, case-control study, Germany, 2020-2021. Data set limited to COVID-19 wave 3 in Germany (see Reference 7) (325 cases, 1083 controls).** Each single exposure variable was analysed in a logistic regression model with 5 adjusting covariables (age group, sex, urban/rural type of district of residence, vaccination status and 7-day-incidence). Observations with missing values in the variable of interest or any of the 5 adjusting covariables were excluded from analysis.

| **Variable** | **Cases**  **% (n/N)** | **Controls**  **% (n/N)** | **aOR^a^** | **95%-CI^b^** | ***P*-value** |
| --- | --- | --- | --- | --- | --- |
| **Pre-existing medical condition** |  |  |  |  |  |
| High blood pressure | 21.1% (68/322) | 21.7% (234/1079) | 1.21 | 0.87–1.68 | 0.266 |
| Other cardiovascular disease | 5.3% (17/322) | 7.2% (78/1077) | 0.81 | 0.46–1.41 | 0.451 |
| Diabetes | 3.7% (12/322) | 6.0% (65/1077) | 0.69 | 0.36–1.32 | 0.260 |
| Pulmonary disease, e.g., asthma, chronic bronchitis, COPD, lung emphysema) | 15.9% (51/321) | 8.6% (93/1077) | 2.11 | 1.45–3.08 | <0.001 |
| Weakened immune system (due to disease or medication) | 5.0% (16/321) | 4.8% (51/1072) | 1.24 | 0.69–2.23 | 0.479 |
| **Overweight (BMI>25)^c^** | 51.6% (166/322) | 53.1% (574/1081) | 1.05 | 0.80–1.36 | 0.740 |
| **Smoking tobacco products^d^** | 16.8% (54/322) | 21.5% (232/1080) | 0.69 | 0.50–0.97 | 0.031 |
| **Frequency of smoking tobacco products** |  |  |  |  |  |
| Smokes daily | 12.1% (39/322) | 15.9% (172/1080) | 0.68 | 0.46–1.01 | 0.058 |
| Smokes occasionally | 4.7% (15/322) | 5.6% (60/1080) | 0.67 | 0.36–1.22 | 0.188 |
| Smoked in the past | 24.5% (79/322) | 25.4% (274/1080) | 0.93 | 0.69–1.27 | 0.658 |
| Never smoked | 58.7% (189/322) | 53.2% (574/1080) | Reference |  |  |
| **Vaping (e-cigarettes or similar)** | 3.4% (11/322) | 2.5% (27/1081) | 1.24 | 0.60–2.57 | 0.552 |

^a^adjusted odds ratio (model with variables age group, sex, urban/rural type of residency, vaccination status and 7-day-incidence)

^b^95%-confidence interval

^c^versus BMI ≤25

^d^smokes daily or occasionally vs. smoked in the past or never smoked
